# Retinoids, anxiety and peripartum depressive symptoms among Chinese women: a prospective cohort study

**DOI:** 10.1186/s12888-017-1405-0

**Published:** 2017-08-01

**Authors:** Yingchun Zeng, Yingtao Li, Huaan Xia, Shenglan Wang, Jingxuan Zhou, Dunjin Chen

**Affiliations:** 1 Research Institute of Gynecology and Obstetrics, The Third Affiliated Hospital of Guangzhou Medical University, Guangzhou, 510150 China; 2 Zhujiang Hospital of Southern Medical University, Guangzhou, China; 3 Department of Obstetrics, Qingdao Municipal Hospital, Qingdao, China

**Keywords:** Retinoids, Retinol-binding protein, Peripartum depression, Chinese women

## Abstract

**Background:**

The current study aimed to investigate whether serum RBP levels can be a key predictor of peripartum depression (PPD).

**Methods:**

This was a prospective cohort study, conducted at a general teaching hospital in South China. Research participants were evaluated at three time points: the third trimester of pregnancy (T1), after delivery at week one (T2), and after delivery week six (T3) using a set of self-reported questionnaires and blood sample assays.

**Results:**

A total of 156 subjects were included for data analysis. The prevalence of anxiety symptoms ranged from 32.69% to 36.53%. The prevalence of PPD was also high and ranged from 27.56% to 35.89%. In the third trimester, significant predictors of depressive symptoms include serum retinol-binding protein (RBP) concentrations and estradiol levels (*P* = 0.008 and 0.033, respectively). At one week after delivery, serum concentrations of RBP at T2 were still significant predictors of depressive symptoms (*P* = 0.020, and serum estradiol concentrations at T1 were a significant predictor (*P* = 0.010). The most stable predictor of depressive symptoms at T3 was anxiety symptoms, especially at T3 time point (*P* < 0.001). Serum RBP concentrations at T1 and T2 were still significant predictors of depressive symptoms at T3.

**Conclusion:**

A high prevalence of anxiety and depressive symptoms tended to persist in Chinese women during the peripartum period. This study, which found the potential contribution of RBP to the occurrence of PPD, requires that large sample studies be conducted in future with a longer-term follow-up period, in order to confirm its results.

## Background

Depression is the leading cause of disease-related disability in women globally [1]. Peripartum anxiety and depression are the most common complications of childbearing, and are associated with substantial adverse effects on pregnancy outcomes, such as limiting a woman’s ability to function effectively in the maternal role [2–4]; as well as with adverse effects on the developing child in terms of preterm delivery, lower birth weight and later cognitive, affective and behavioral disturbances [4, 5]. Consequences of depression and anxiety are often more severe in the peripartum period than during other life periods [6]. A number of studies suggest that prevalence rates of anxiety symptoms range from 25% to 45%, and are increasingly common during the peripartum period [4, 7]. Prevalence of peripartum depression (PPD) is up to 25% around the world [8]. In China, the prevalence of PPD varies between 28.5% and 38.8% [9, 10]. Given the high prevalence and severe consequences of PPD, there is a need for early detection and prevention strategies for women with PPD.

Retinoids, Vitamin A and its derivatives, have been linked to neuropsychiatric symptoms including depression [11]. Excess accumulation of Vitamin A has been reported to induce depression [12]. Mawson and Wang [13] proposed a theory of retinoid toxicity hypothesis of PPD: Vitamin A accumulates in breast, brain and liver to potentially toxic concentrations in the third trimester. Increased levels of Vitamin A may impair the mobilization and secretion of retinol-binding protein (RBP), which may cause PPD by increasing the levels of circulating retinoic acids and retinyl esters [13]. Serum RBP estimation highly correlates with retinol concentration to predict Vitamin A status [14]. Hence, measuring retinoids by serum RBP concentrations may be a sensitive and specific predictor or biomarker of PPD in women. Therefore, this study aimed to investigate whether serum RBP levels can be a putative biomarker of PPD. This study hypothesized that serum RBP concentrations might be a key predictor of PPD, and could be used as a putative biomarker for the early detection and diagnosis of PPD.

## Methods

### Study design and procedure

This was a prospective cohort study, conducted at a general teaching hospital in South China. Research participants were evaluated at three time points: the third trimester of pregnancy (T1), after delivery at week one (T2), and after delivery week six (T3) using a set of self-reported questionnaires and blood sample assays. This study was approved by the Ethical Review Committee of the Third Affiliated Hospital of Guangzhou Medical University. Prior to subject recruitment and data collection, the researcher provided a full explanation of the study purpose and procedure. All research participants, who voluntarily joined this study, gave written informed consent. This study adhered to STROBE methodology.

### Participants

Women were recruited at an outpatient obstetric clinic from January to August 2016. Inclusion criteria were pregnant women ages 18 years or older, who were planning to give birth and attend postpartum follow up at the studied hospital, with normal singleton pregnancies. Exclusion criteria included women with severe mental health problems, such as psychiatric disease, or physical illnesses requiring medical treatment or admission to hospital.

### Measures

Demographic and clinical variables included age, marital status, highest level of education, monthly income, parity (first birth or subsequent), delivery week, delivery mode (vaginal birth or Caesarian section), and mode of infant feeding (exclusive breastfeeding rates). Self-report measures were collected to characterize participants’ sleep, anxiety and depressive symptoms. Previous research found that women with symptoms such as anxiety and sleep disturbance were significantly correlated with PPD [15], so this study also included sleep quality and anxiety symptom measures.

The Pittsburgh Sleep Quality Index (PSQI) was utilized to evaluate subjects’ sleep quality. The PSQI consists of 18 items composed of seven factors; each factor is scored by 0–3 points and the total score ranges from 0 to 21 points; a higher score indicates worse sleep quality [16]. The Chinese version of PSQI has been validated in a Chinese population, and has established good reliability and validity [16, 17]. Anxiety symptoms were assessed using the Self-rating Anxiety Scale (SAS), originally developed by Zung [18]; SAS is a 20-item Likert-style (4-point) rating scale for anxiety, with a theoretical score range extending from 20 to 80. The original total score plus 1.25 will be the “Anxiety Index” score (<50 as normal; ≥50 as anxiety cases) [19]. The Chinese version of SAS is also widely used among Chinese women during pregnancy [20]. In this study, the internal consistency by Cronbach’s alpha of SAS was 0.89. The Edinburgh Postnatal Depression Scale (EPDS) is a widely used depression assessment scale for peripartum women globally [21]. It has 10 items, with each item divided into 0–3 points; the total score ranges from 0 to 30 points [21]. The Chinese version of the EPDS has been validated in a sample of Chinese women in the perinatal period. The cut-off score of 9/10 can show sensitivity with 82% and specificity with 86% at six weeks postpartum [22]. Hence, subjects in this study with an EPDS score ≥ 10 were taken as “peripartum depression” cases. Studies have shown the scale has good reliability and validity in populations in mainland China [23, 24].

### Laboratory investigation

Blood samples for serum RBP assays were obtained in late pregnancy (30–34 weeks of gestation), and at one week and six weeks after delivery. Blood draw was performed in the morning within two hours (8 a.m. to 10 a.m.). The serum levels of RBP were assessed by human ELISA (Enzyme-Linked Immunosorbent Assay) kits for research use only (Abcam Trading Company Ltd., Guangzhou, China). Serum was frozen until assayed. The intra- and inter-assay coefficient of variation averaged 4.5% and 7.2%, respectively. The normal reference value of RBP used was 25–80 mg/L. During the peripartum period, the tremendous changes in gonadal hormones, such as estrogen and progesterone, could be triggers for depression [25]. Hence, laboratory determinations of serum concentrations of estradiol and progesterone were also carried out by commercially available kits using RIA (radioimmunoassay). The coefficients for intra-assay and inter-assay variation were 4.3% and 6.8% for estradiol and 5.1% and 8.8% for progesterone [26]. For both hormones, all samples were analyzed in a single batch.

### Statistical analysis

Statistical analyses were performed using SPSS Version 21.0 and AMOS 21.0. Descriptive analysis of continuous and categorical variables was performed. Continuous variables were expressed as means ± SD, and categorical variables were expressed as proportions (%) by SPSS 21.0. Correlation analysis was used to identify potential predictors for EPDS score at each time assessment. Those variables with *p* values less than 0.05 were entered into the regression models. In order to control any potential collinear relation of variables, SEM (Structural Equation Modeling) regression analysis was used to identify predictors of depressive symptoms in women in the peripartum period by AMOS 21.0. A *p*-value of 0.05 was considered to be statistically significant.

## Results

Of 200 potential eligible patients approached, 181 agreed to join this study. Among those who declined to join this study, 14 were too busy, and five were planning to give birth in a different hospital, and so would be unable to take part in the follow-up visits. Among the 181 subjects, only 156 completed two follow-up assessments after delivery at week one and six. Hence, a total of 156 subjects were included for data analysis.

Table 1 shows the socio-demographic and clinical data. All were married, with the mean age of participants 26.65 (Standardized Deviation-SD, 2.7), with ages ranging from 22 to 35 years old. Most had high levels of education (*n* = 134, 87%), at the undergraduate level or above. Nearly three-quarters of the women had monthly income varying from 3000 to 5000 RMB (approximately $500 to $833 U.S.). More than 60% (*n* = 97, 63%) were primiparous. The mean of delivery week was 38.99 (SD, 1.31), ranging from 36 to 41 weeks. More than half (*n* = 84, 54.5%) had a vaginal delivery. Nearly one-third exclusively breastfed at T1 (*n* = 52, 33.33%), and less than one-half of women exclusively breastfeed at T2 (*n* = 76, 48.71%).

**Table 1:** Descriptive statistics of variables used in the analysis (*N* = 156)

Variables	Mean (SD)	n (%)
Age (years)	26.65 (2.70) (22 to 35)	
Education level		
High school or below		7 (4.50)
College		13 (8.40)
Undergraduate or high		134 (87.0)
Monthly income (RMB)		
< 3000		10 (6.50)
3000–5000		114 (74.0)
> 5000		30 (19.50)
Parity		
First birth		97 (63.0)
Subsequent		57 (37.0)
Weeks of delivery	38.99 (1.31) (36 to 41)	
Delivery mode		
Vaginal birth		84 (54.50)
Cesarean section		70 (45.50)
Exclusive breastfeeding (T2)		52 (33.33)
Exclusive breastfeeding (T3)		76 (48.71)
RBP level (mg/L)		
T1	38.39 (2.47)	
T2	40.35 (1.62)	
T3	36.86 (1.97)	
Estradiol level (pmol/L)		
T1	14,539.46 (1503.49)	
T2	2790.27 (1081.80)	
T3	231.76 (49.89)	
Progesterone level (nmol/L)		
T1	205.81 (52.53)	
T2	12.58 (8.95)	
T3	4.72 (1.82)	
Mean PSQI score		
T1	8.67 (3.48)	
T2	8.79 (3.55)	
T3	8.61 (3.35)	
SAS score		
≥ 50 at T1		54 (34.61)
≥ 50 at T2		51 (32.69)
≥ 50 at T3		57 (36.53)
EPDS score		
≥ 10 at T1		48 (30.76)
≥ 10 at T2		43 (27.56)
≥ 10 at T3		56 (35.89)

T1, at the third trimester; T2, after delivery at week one; T3, after delivery at week six

*Abbreviations: EPDS* Edinburgh Postnatal Depression Scale, *PSQI* Pittsburgh Sleep Quality Index, *RBP* retinol-binding protein, *SAS* Self-rating Anxiety Scale

A summary of descriptive statistics of psychosocial variables, RBP and reproductive hormones is also shown in Table 1. Serum RBP concentrations were lowered at T3, serum estradiol and progesterone concentrations dropped sharply from T1 to T3. Sleep quality remained relatively stable. The prevalence of anxiety symptoms was relatively high, ranging from 32.69% to 36.53%. The prevalence of depressive symptoms reached the highest level at T3 (35.89%), followed by the period of late pregnancy (30.76%).

By SEM regression analysis, predictors of depressive symptoms at each time point were shown in Tables 2, 3, and 4. In the third trimester, significant predictors of depressive symptoms include serum RBP concentrations and estradiol levels (*P* = 0.008 and 0.033, respectively). Anxiety symptoms significantly predict depressive symptoms (*P* = 0.022) (Table 2). At one week after delivery, serum concentrations of RBP at T2 were still significant predictors of depressive symptoms (*P* = 0.020), serum estradiol concentrations at T2 were not a significant predictor (*P* = 0.346), and serum estradiol concentrations at T1 were a significant predictor (*P* = 0.010). Anxiety symptoms at T1 and T2 both significantly predicted depressive symptoms (*P* = 0.027 and 0.015, respectively) (Table 3). From Table 4, the most stable predictor of depression at T3 was anxiety symptoms, especially at T3 time point (*P* < 0.001). Serum RBP concentrations at T1 and T2 were significant predictors of depressive symptoms at T3.

**Table 2:** Regression coefficients of predictors for EPDS score at T1

	Estimates	SE	CR^a^	P
T1_EPDS score ← Education	0.041	0.194	1.408	0.159
T1_EPDS score ← RBP_T1	−0.642	0	−2.684	0.008
T1_EPDS score ← Estradiol_T1	−0.066	0.005	−2.130	0.033
T1_EPDS score ← Progesterone_T1	0.051	0.011	1.224	0.221
T1_EPDS score ← PSQI_T1	0.049	0.096	0.478	0.633
T1_EPDS score ← SAS_T1	0.395	0.065	2.282	0.022

^a^CR, Critical ratio (the estimate divided by its standard error). T1, at the third trimester

*Abbreviations: EPDS* Edinburgh Postnatal Depression Scale, *PSQI* Pittsburgh Sleep Quality Index, *RBP* retinol-binding protein, *SAS* Self-rating Anxiety Scale

**Table 3:** Regression coefficients of predictors for EPDS score at T2

	Estimates	SE	CR^a^	P
T2_EPDS score ← Education	−0.029	0.216	−0.870	0.385
T2_EPDS score ← Income	−0.001	0.215	−0.043	0.966
T2_EPDS score ← RBP_T1	0.062	0	1.609	0.108
T2_EPDS score ← Estradiol_T1	−0.083	0.651	−2.576	0.010
T2_EPDS score ← Progesterone_T1	−0.030	0.009	−0.220	0.826
T2_EPDS score ← PSQI_T1	0.136	0.097	1.313	0.189
T2_EPDS score ← SAS_T1	0.244	0.035	2.205	0.027
T2_EPDS score ← RBP_T2	−0.766	0.001	−2.138	0.020
T2_EPDS score ← Estradiol_T2	0.046	1.146	0.942	0.346
T2_EPDS score ← Progesterone_T2	−0.142	0.060	−0.858	0.391
T2_EPDS score ← PSQI_T2	0.077	0.096	0.739	0.460
T2_EPDS score ← SAS_T2	0.397	0.054	2.442	0.015

^a^CR, Critical ratio (the estimate divided by its standard error). T2, after delivery at week one

*Abbreviations: EPDS* Edinburgh Postnatal Depression Scale, *PSQI* Pittsburgh Sleep Quality Index, *RBP* retinol-binding protein, *SAS* Self-rating Anxiety Scale

**Table 4:** Regression coefficients of predictors for EPDS score at T3

	Estimates	SE	CR^a^	P
T3_EPDS score ← Income	0.036	0.207	1.193	0.233
T3_EPDS score ← Exclusive breastfeeding	−0.041	0.343	−1.320	0.187
T3_EPDS score ← RBP_T1	−0.538	0.071	−2.952	0.003
T3_EPDS score ← Estradiol_T1	−0.232	0.009	−1.758	0.079
T3_EPDS score ← Progesterone_T1	0.002	0.004	0.081	0.935
T3_EPDS score ← PSQI_T1	0.123	0.093	1.289	0.197
T3_EPDS score ← SAS_T1	0.090	0	2.481	0.013
T3_EPDS score ← RBP_T2	−0.543	0.053	−3.571	<0.001
T3_EPDS score ← Estradiol_T2	0.059	0.073	1.442	0.149
T3_EPDS score ← Progesterone_T2	0.019	0.061	0.119	0.905
T3_EPDS score ← PSQI_T2	0.184	0.096	1.838	0.066
T3_EPDS score ← SAS_T2	0.268	0.010	2.066	0.039
T3_EPDS score ← RBP_T3	−0.325	0.910	−1.335	0.182
T3_EPDS score ← Estradiol_T3	−0.005	0.071	−0.13	0.896
T3_EPDS score ← Progesterone_T3	0.189	0.008	1.573	0.116
T3_EPDS score ← PSQI_T3	0.044	0.422	0.902	0.367
T3_EPDS score ← SAS_T3	0.585	0.051	4.074	<0.001

^a^CR, Critical ratio (the estimate divided by its standard error). T3, after delivery at week six

*Abbreviations: EPDS* Edinburgh Postnatal Depression Scale, *PSQI* Pittsburgh Sleep Quality Index, *RBP* retinol-binding protein, *SAS* Self-rating Anxiety Scale

## Discussion

The prevalence of PPD ranged from 27.56% to 35.89% in this study sample. Anxiety symptoms were also very common, with more than one-third of women reporting anxiety symptoms during the peripartum period. Symptoms of anxiety and depression tended to persist during the peripartum period. Compared with one in seven women reporting PPD in the United States [15], Chinese women in this study experienced a far higher prevalence of PPD, and urgently need more intervention strategies to treat and control depressive symptoms. This study also found a high prevalence of anxiety, so Chinese women in the peripartum period should be screened for these two common symptoms in a routine clinical setting. A routine screening strategy may help childbearing women prevent PPD and achieve better mental health and pregnancy outcomes. Consistent with previous research [7], there were no socio-demographics or obstetric variables that significantly predicted PPD.

This study partly supported the theory of retinoid toxicity hypothesis of PPD proposed by Mawson and Wang [13]. Serum RBP levels were significant predictors of PPD during periods in late pregnancy and the first week after delivery, but at six weeks after delivery, there was a non-significant predicting relationship between RBP levels and EPDS scores. Mawson and Wang [13] proposed that vitamin A accumulates in breast, brain and liver to potentially toxic concentrations in the third trimester, such that increased liver concentrations of vitamin A impair the mobilization and secretion of RBP, which may cause PPD by increasing the levels of circulating retinoic acids and retinyl esters. This study found that serum RBP estimation correlates highly with retinol concentration, so that measuring retinoids by serum RBP concentrations could be a sensitive and specific predictor or biomarker of PPD in women. Consistent with Mawson and Wang’s hypothesis, RBP was strongly associated with PPD symptoms. This study did not, however, assess retinyl esters or retinoic acid. Thus, future work should investigate the possible role of other vitamin A metabolites in PPD.

Within this retinoid toxicity theory of PPD, Mawson and Wang [13] proposed that breastfeeding could reduce body stores of Vitamin A for women and prevent the occurrence of PPD. Although previous research evidence also indicates a strong association between breastfeeding and PPD [27], the present study did not find a significant predicting relationship between exclusive breastfeeding and EPDS score. One possible explanation may be due to the lower rates of exclusive breastfeeding and short-term follow-up assessment after six weeks of delivery. Therefore, whether breastfeeding could prevent PPD by reducing maternal body stores of Vitamin A needs further investigation.

This study found that the role of gonadal hormones, such as estradiol at T1, were significant predictors of PPD at T1 and T2. As women within one week after delivery, it is a time when their gonadal hormones fluctuate dramatically, and result in a significant predicting relationship between estradiol and the occurrence of PPD [28]. In addition, this study’s findings were consistent with previous empirical research [6, 29], with anxiety levels significantly predicting PPD at three assessment points. Hence, relevant intervention programs should be developed in order to help women improve their rate of breastfeeding, and reduce anxiety levels.

There were limitations that may have impacted the interpretations of the study findings. Most of the women in this sample were well-educated, and all were married. Hence, this study’s findings exclude the experiences of single mothers and those with lower levels of education. Additionally, hormone levels were only assayed for estradiol and progesterone; other hormones, such as prolactin, luteinizing, and follicle stimulating hormones should also be assayed in future studies, to examine their possible relationship with PPD. A further limitation of the present study is the relatively short-term follow-up period and relatively small sample size. Thus, larger sample studies with longer-term follow-up periods are required, in order to address these limitations.

## Conclusions

This study found that a high prevalence of anxiety and depressive symptoms tended to persist in Chinese women during the peripartum period. This study, which also found the potential contribution of RBP to the occurrence of PPD, requires that large sample studies be conducted in future with a longer-term follow-up period, in order to confirm its results. In addition, there is a need for future work to investigate the possible role of other vitamin A metabolites in PPD, i.e., retinyl esters, the percentage of retinyl esters is higher than 10% of total vitamin A - an indicator of retinoid toxicity - and the concentration of retinoic acid, as per the retinoid toxicity hypothesis of PPD proposed by Mawson and Wang [13].
